# Evaluation of methanol content of illegal beverages using GC and an easier modified Chromotropic acid method; a cross sectional study

**DOI:** 10.1186/s13011-019-0244-z

**Published:** 2019-12-16

**Authors:** Nasim Zamani, Ali Rafizadeh, Hossein Hassanian-Moghaddam, Alireza Akhavan-Tavakoli, Mahdi Ghorbani-Samin, Maryam Akhgari, Shahab Shariati

**Affiliations:** 1 Social Determinants of Health Research Center, Shahid Beheshti University of Medical Sciences, Tehran, Iran; 2 Department of Clinical Toxicology, Loghman Hakim Hospital, Shahid Beheshti University of Medical Sciences, South Kargar Street, Tehran, Iran; 3 Departments of Nursing & Midwifery, Rasht Branch, Islamic Azad University, Rasht, Iran; 4 Legal Medicine Research Center, Legal Medicine Organization, Rasht, Iran; 5 Department of Forensic Toxicology, Legal Medicine Research Center, Legal Medicine Organization, Tehran, Iran; 6 Department of Chemistry, Rasht Branch, Islamic Azad University, Rasht, Iran

**Keywords:** Methanol, Modified chromotropic acid method, Alcoholic beverages

## Abstract

**Background:**

Methanol is highly toxic to human beings and naturally exists in some beverages. Having access to an easy and cheap method for its determination is of great importance to increase the safety of use of these beverages. Our main aim is to evaluate methanol concentration of some alcoholic beverages in Iran black market and compare it with the European and US standards. Also, we evaluated the efficacy of a newly designed and produced chemical kit in determining the risk of methanol toxicity by drinking of such samples compared to gas chromatography method.

**Methods:**

Methanol content of suspected alcoholic beverages referred to forensic toxicology laboratory, Guilan province, Iran was measured using gas chromatography and a recently designed kit based on modified colorimetric chromotropic acid method.

**Results:**

Of 1221 samples, 145 (11.9%) had no ethanol content, while in three samples (0.25%), methanol was high enough (700,000; 870,000; 920,000 mg/L) to cause severe methanol toxicity. Median [IQR] ethanol content of the suspected samples was 9% [3.7, 32.75]. Methanol was detected in 128 (10.48%) samples using gas chromatography method and 160 samples (13.1%) with designed kit with 100% sensitivity, 97.07% specificity, and 100% negative-predictive-value.

**Conclusions:**

Alcoholic beverages produced in local black market in Iran are not safe at all. The application of the new method is practical, rapid, easy, and accurate to evaluate the risk of methanol toxicity in suspected alcoholic drinks.

## Introduction

Toxic alcohol consumption is a major cause of mortalities and morbidities worldwide [1]. Although drinking alcohol is prohibited in Muslim countries and there have been major penalties determined for alcohol use in them, recent statistics show that these penalties have failed to decrease the frequency of alcohol use or misuse in some of them [2]. This has resulted in increased use of black market alcohol which may potentially be methanol-contaminated due to the lack of observatory quality control processes and outbreaks of methanol poisoning in different parts of the world [3, 4]. Considering the Eastern Mediterranean Region (EMR) as the region with Islamic countries within, both men and women in this area have the highest weekly heavy episodic drinking among drinkers in the past 12 months in both males and females worldwide [1].

The worldwide consumption of ethanol was equal to 6.13 l of pure alcohol consumed per person of 15 years of age or older in 2005. A large portion of this consumption – 28.6% or 1.76 l per person – was homemade, illegally produced or sold outside normal government controls [1]. This increases the risk of introduction of hazardous chemicals into the ethanol, the most important of which is methanol [5]. Both unsupervised production of alcoholic beverages and lack of quality control processes during their production increase the risk of contamination of the produced alcohol with unwanted toxic components including methanol. Therefore, during the process of quality control of production of such beverages, it is generally important to be able to determine the presence of sufficient methanol concentration capable of resulting in poisoning.

Police were usually asked to investigate the discovered consignment of suspected alcoholic beverages and report its content to the judiciary system to determine their alcohol concentration. Based on Iranian legal medicine organization protocols, liquids with 3% v/v ethanol or less than that are not legally considered to be alcoholic beverage at all.

The gold standard method for determination of methanol content in alcoholic beverages is gas chromatography (GC). However, this technique is expensive, calls for considerable knowledge and experience to be performed, and is not readily available in many developing countries although this technique has previously been used even in mass poisonings [5]. Having access to a safe, cheap and easy method to prove the absence of unauthorized quantities of methanol before ingestion is therefore highly advantageous [6].

Generally, with the same methanol concentration, the possibility of toxicity increases with reduced ethanol content. Ethanol has a 20 times higher affinity for liver alcohol dehydrogenase enzyme which prevents methanol metabolism when blood ethanol level is 100 mg/dL or higher [4]. Previous studies declare up to 5 mg/dL serum methanol level as the acceptable concentration of this toxic agent in human blood [7]. Reaching this methanol level in an average 75-kg adult with about 41 l of body water (55% of the total body weight) would roughly be possible after consuming 251 mg methanol in 1–2 h. This is approximately equal to 2.5% v/v absolute methanol in water [7]. Thus, determination of the maximum acceptable methanol to ethanol concentration in an alcoholic drink without risking toxicity is a challenging concern. The “maximum safe” concentration of methanol in alcoholic beverages has previously been determined based on “permitted and safe content of methanol in the beverages” regulated by the European Parliament and the Council (4000 mg/L in alcoholic drinks with 40% v/v ethanol concentration) and US national research council of the national academies (Table 1) [8, 9]. Therefore, “maximum safe dose” is defined to avoid a serum methanol concentration more than 5 mg/dL [5, 8–11].

**Table 1:** Methanol Concentrations in Food and Beverages

Source (average ETH%)	Concentration (mg/L)
Fresh and canned fruit juices (orange and grapefruit juices) (< 0.5%)	1–43 11–80 12–640 (Average of 140)
Beer (4–8%)	6–27
Wines (9–16%) Fortified wines (16–24%)	96–329
Distilled spirits (36–50%)	16–220
Brandies (35–60%)	6000-7000
Neutral spirits (85–95%)	< 1500

We used a new kit designed based on the modified chromotropic acid (CA) method for this purpose. Using this kit, the relative concentration of methanol to ethanol is estimated since methanol/ethanol ratio can predict the potency of the drink to induce methanol toxicity. Therefore, a positive test would indicate an unsafe beverage and the possibility of methanol poisoning. We picked a conservative approach to evaluate the potency for both acute and chronic methanol toxicities. The table for safe concentration of methanol in different food products and beverages (USA standard) was therefore used (Table 1) [8–11] which determined all drinks with any concentration below the permitted levels as safe beverages. Preliminary evaluations confirmed the efficacy of this kit in determination of possible toxicity risk of the alcoholic beverages [12].

The aim of the current study was to firstly evaluate the methanol and ethanol contents of the suspected alcoholic beverages discovered by Iranian police as sample of the alcoholic beverages available in the Iranian black market using GC as the gold standard method. As a second aim, we assessed the potency of toxicity of these suspected samples by detection of relative methanol to ethanol content using a new kit based on modified CA method and compared them with the results obtained by GC in order to determine the efficacy of the designed kit.

## Methods

Between March 2017 and May 2018, Guilan office, Legal Medicine Organization (LMO) analyzed the methanol and ethanol contents of more than one-thousand suspected alcoholic beverage samples referred by police using a gas chromatography apparatus (Yanglin model: YL 6100 -South Korea). The newly designed kit produced by Arya Mabna Tashkhis Co., Tehran, Iran was used to detect the potency of induction of methanol poisoning by qualitative detection of the relative methanol to ethanol contents in the samples. This kit contained five reactants (shown by A, B, C, D and E), a calibrated standard color strip which was used to give a better interpretation of gained results as potency for toxicity of the beverages, and an instruction brochure. The obtained results by both techniques were compared together. Technicians in each section (GC and kit interpretations) were blind to the results obtained by other divisions.

### Procedure of the GC method

The GC instrument used in this study was a Yanglin model: YL 6100 (South Korea). GC system was equipped with a flame ionization detector (FID) and Tr_2b-5_. The length and inner diameter of Si capillary columns were 30 m and 0.53 mm, respectively. Helium carrier gas (flow rate = 4 mL/min) was used as carrier gas for methanol separation. All standards and samples were directly injected (2 μL) to GC system (with split ratio 1:20) with column temperature pre-incubated at 80 °C as isothermal. The oven, injector and detector temperatures were fixed at 80, 240 and 280 C degrees, respectively. To evaluate the methanol and ethanol contents using this device, a 320-mg/dL (3200 mg/L) standard concentrations of methanol and ethanol by Merck were used [13].

### Methanol analysis using proposed kit

Fifty microliters of each sample was drawn into a clean test tube. Fifty μL of the reactants A (sulfuric acid solution) and B (potassium permanganate) was added to the tube to oxidize the methanol to formaldehyde and formic acid respectively. In this step due to presence of high amounts of Mn^7+^ and reducing agents, the color of solution changed from deep purple to brown. After three minutes, 50 μL of the reactant C (sodium hydrogen sulfite) was added to the test tube and (the mixture) shaken thoroughly to get a completely colorless solution. Then, 50 μL of reactant D (chromotropic acid) and one mL of reactant E (concentrated sulfuric acid) were added and shaken. In this step, formic acid is reduced to formaldehyde and reacts with its specific color indicator (chromotropic acid) that is accompanied with the stable violet complex. The intensity of the appeared color depends on the relative ethanol/methanol concentration. A maximum wait time of 5 min was made and the color change was read comparing the test color with the standard reference color strip, calibrated to the European standard, to obtain positive (not safe drink) or negative (safe drink) results. Safe drinks were considered as those with less than the EU cutoff of methanol (4000 mg/L in a 40% alcohol) and not only based on the methanol content, per se.

### Statistical analysis

Statistical analysis was done by statistical package for social sciences (SPSS) version 24 (IBM Corporations, Chicago, Ill, USA) using Pearson Chi square and McNemar test. Simple descriptive analysis was done using median [IQR] and mean ± SD or frequency (%). Sensitivity, specificity, positive predictive value (PPV), negative predictive value (NPV), accuracy and prevalence of kit compared to gold standard using 95% confidence interval (CI). A *P* value less than 0.05 was considered to be statistically significant.

## Results

**Fig. 1:**
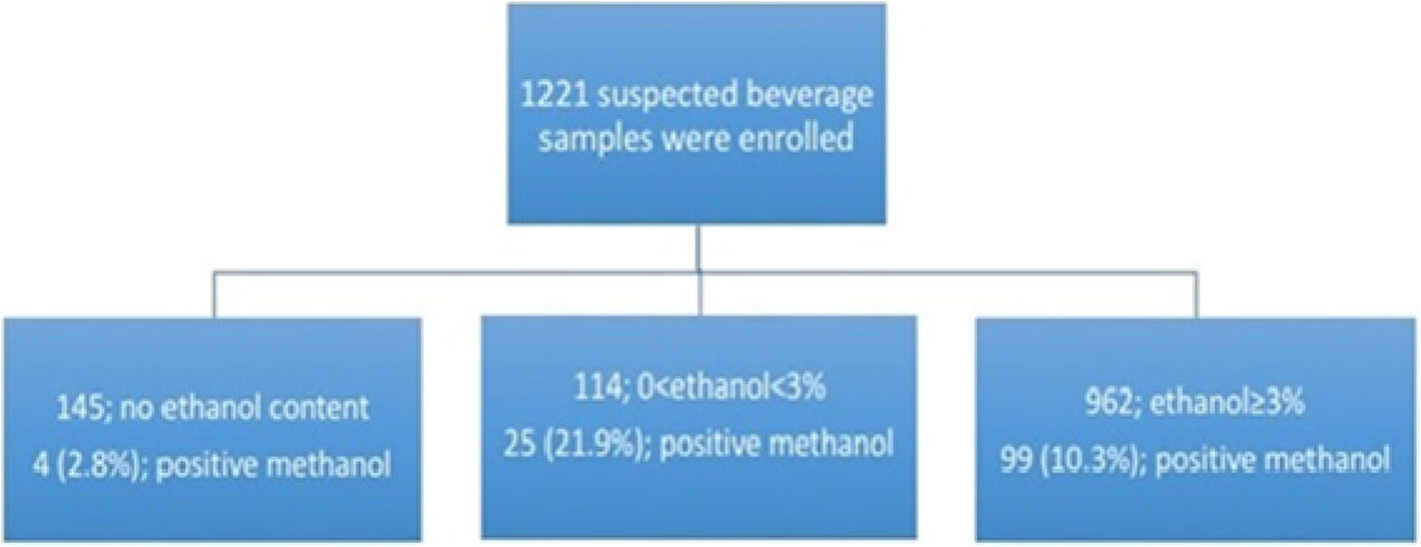
Sample recruitment

**Fig. 2:**
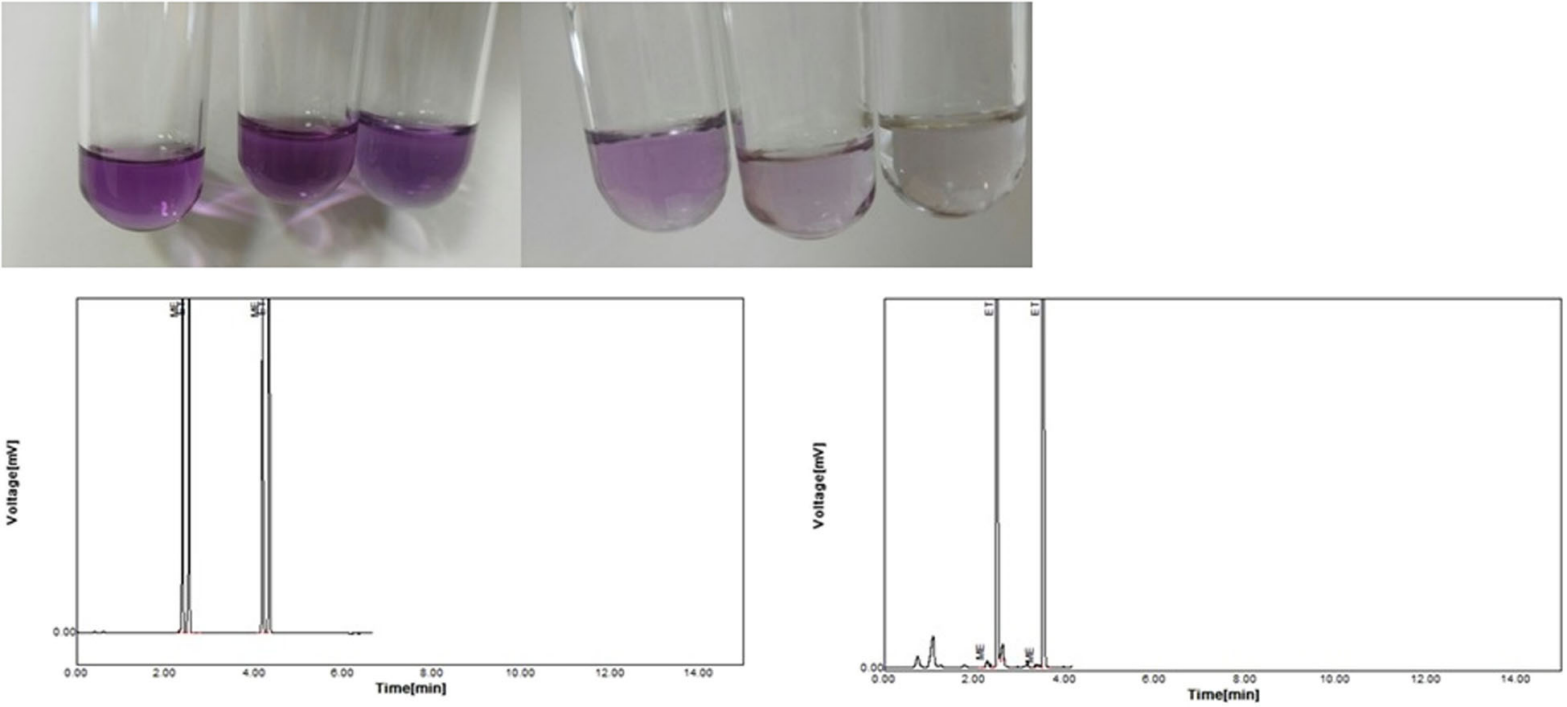
Positive (left) versus negative (right) test results

A total of 1221 samples were referred to Guilan forensic medicine lab by police during the study period. Of them, 145 (11.9%) had no ethanol content. Ethanol content was equal to or less than 3% v/v in 114 and more than that in 962 samples (Figs. 1 and 2). Median [IQR] ethanol content was 20,000 [15,000- 25,000] mg/L in the first group (≤3% EtOH; 114 samples) and 130,000 [70,000- 370,000] mg/L in the second group (> 3% EtOH; 962 samples). The median [IQR] ethanol content of the suspected samples was 9% [3.7, 32.75]. In three samples (0.25%) methanol was high enough (700,000; 870,000; and 920,000 mg/L) to cause severe methanol toxicity in consumers. Methanol was detected in 128 (10.48%) samples by GC method (range 8.5 to 920,000 mg/L) and 160 samples (13.1%) with designed calorimetric kit (100% sensitivity (95% CI; 97.17–100), 97.07% specificity (95% CI; 95.89–97.99) and 100% negative predictive value. Table 2 shows the comparison of the results withdrawn by GC (gold standard method) and newly designed kit. It also shows the safety of the beverages based on the kit results. Table 3 shows the quantitative methanol and ethanol contents of the discovered alcoholic beverages (determined by GC) during the study period. Accordingly, the median [IQR] (min, max) volume needed for producing a methanol serum level of 20 mg/dL for an average 70-kg Iranian was 57.74 [34.46, 103.46] (0.009, 988.23) liters.

**Table 2:** Qualitative Diagnostic Characteristics of Methanol Content of Suspected Beverages Using Modified CA Method and Gold Standard Gas Chromatography (*n* = 1221, *p* < 0.001)

		Gold standard		Sensitivity (95% CI)	Specificity (95% CI)	PPV (95% CI)	NPV (95% CI)	Accuracy (95% CI)	Prevalence (95% CI)
Test Results		positive	negative						
	positive	128	32	100 (97.16–100)	97.07 (95.89–97.99)	80 (73.98–84.91)	100 (100)	97.38 (96.32–98.2)	10.48 (8.82–12.34)
	negative	0	1061						
		Safe drink							
	positive	123	37	100 (97.05–100)	96.63 (95.38–97.62)	76.88 (70.78–82.02)	100 (100)	97.86 (95.85–98.2)	10.07 (8.44–11.09)
	negative	0	1061

**Table 3:** Quantitative Ethanol and Methanol Contents of Suspected Referred Samples during 14 months (*n* = 1221)

	No ethanol (*n* = 145)	Ethanol< 30,000 mg/L (*n* = 114)	Ethanol≥30,000 mg/L (*n* = 962)	Total Ethanol mg/L (n = 1221)
Median [IQR] Methanol (mg/L)	0	0	0	0
Median [IQR] Ethanol (mg/L)	0	20,000 [15,000- 25,000]	130,000 [70,000; 370,000]	90,000 [37,000- 327,500]
Mean ± SD Methanol (mg/L)	17,175 ± 119,330	34.53 ± 105.18	19.16 ± 74.62	2058 ± 41,371
Mean ± SD Ethanol (mg/L)	0	19,380 ± 6634	177,190 ± 197,485	177,190 ± 197,485
Methanol range (mg/L)	(0–920,000)	(0, 829)	(0, 924)	(0–920,000)
Ethanol range (mg/L)	0	(1000–29,000)	(30,000- 970,000)	(0–970,000)

## Discussion

Methanol, a potent toxicant in humans, occurs naturally at a low level in most alcoholic beverages without causing harm [6]. “Unrecorded” alcohol constitutes about 30% of all alcohol globally consumed [12]. It is an over-view category for any alcohol not taxed or registered in the jurisdiction where it is consumed. Due to the relatively limited information and difficulties in measuring this category, its public health consequences are not well described [14, 15]. Illegal alcohol consumption is mostly common in Europe, particularly in Eastern Europe, followed by South America and Africa [16].

Deficiency in determination of the methanol content of beverages is even graver in countries like Iran and Indonesia with Islamic rules, where selling and buying alcoholic beverages are prohibited and there are severe penalties for people who sell and buy it. Previous studies have shown high amounts of methanol and other unwanted chemicals that could potentially cause toxicity in unrecorded alcoholic beverages [17, 18].

Considering the fact that in spite of methanol outbreaks, evaluation of the black market alcoholic beverages is not common in Iran, we decided to evaluate the methanol content (as the most dangerous component in homemade alcohols that can readily cause severe poisoning and even death) of some discovered alcoholic beverages referred to LMO of Guilan, Iran [19].

Lachenmeier and colleagues showed that the majority of their samples (64%) had an alcohol content between 35 and 40% v/v, being in accordance with the typical strength of legal spirits in Europe [20]. In another study, the majority of unrecorded alcohol was homemade samohon with alcoholic strength averaging close to 40% v/v [21]. A limited number of samples, advertised for medical purposes, were identified with high alcoholic strengths (above 60% v/v.). Single samples showed contamination with acetaldehyde and ethyl carbamate above the levels of toxicological concern. The mean ethanol content of our samples was 9% which is significantly less than that in homemade alcohols discovered in other countries. It might be due to the fact that almost 12% of the samples had no ethanol content and were only suspected to be alcoholic beverages by police. Even considering alcoholic beverages, the mean ethanol content was 11% that is far less than in other studies. Although we could not determine the content of other illegal products, toxic material, and heavy metals in our beverage samples, this low content of ethanol emphasizes the lack of control and possibly poor quality of the homemade alcohols in Iran. Considering the higher risk of toxicity in beverages with less ethanol and the same methanol content, this fact may jeopardize health of the consumers in the society and needs further evaluations and legal acts by authorities.

One of our interesting findings is that three samples had high levels of methanol with no ethanol. These drinks are likely to cause acute methanol toxicity as there is no ethanol to act as an antidote (i.e. to reduce the rate of conversion of methanol to very toxic metabolites) and thus reduce the toxicity of methanol. Only 9–12 mL of these drinks are able to cause toxicity and if spread widely, they can probably initiate a methanol outbreak with substantial morbidities and mortalities [22].

In homemade alcoholic beverages, we expect to detect both methanol and ethanol. If the concentration of methanol is higher than that of ethanol, it can be metabolized and produce toxic byproducts after the metabolism of ethanol is completed. Lack of ethanol cannot be explained except by using industrial high concentration of methanol that has been added by mistake (instead of industrial ethanol) or deliberately. Methanol is cheaper and easily available and may therefore be sold accidentally instead of ethanol or intentionally added to beverages to strengthen the effects of alcohol for more profit. But, ingested methanol is potentially toxic after a much smaller dose than ethanol and in spite of hemodialysis may causes serious neurological symptoms and death [23–26].

The designed kit falsely reported 37 samples (2.6%) to be unsafe (false positive; not confirmed by GC). False positive results may be due to existence of different compounds that may interact with chromotropic acid including formic acid, formaldehyde, and 2–4 dichlorophenoxyacetic acid (2,4-D) and its derivatives. 2,4-D is a cheap and effective herbicide which may be unusually used to dry grapes [26, 27]. Possibly, reducing the sensitivity of the designed kit can reduce the number of false positive results.

### Strength and limitations

Previous methods were based on detection of methanol content in alcoholic beverages while the current method is novel since it determines the methanol/ethanol ratio in the same products which is more accurate in prediction of risk of methanol toxicity.

We were not able to measure formic acid, formaldehyde and 2,4-D by GC to clarify false positive results. Positive results in designed kit are not accurate in almost 3% (false positive) due to possible interactions with the applied method that can be due to formic acid, formaldehyde or other confounders. We had no false negative results which is a great advantage of the current kit which is designed to determine the risk of a fatal poisoning.

## Conclusion

It is generally believed that local black market stock of alcoholic beverages is not safe. In Iran, some handmade alcoholic beverages contain low levels of ethanol while some contain extremely high levels of methanol that can result in methanol toxicity and even outbreaks [3]. It should be considered that the limited three contaminated samples may be sold and purchased in large amounts and therefore cause mass poisonings. Also, only 10% of the smuggled alcoholic beverages are discovered by Iranian Police each year. The newly designed modified CA kit can successfully determine the potency of the alcoholic beverages to induce methanol poisoning with efficacy comparable to GC method but easier, faster and cheaper. Positive results with the designed kit (unsafe drinks) were confirmed in more than 97% of the cases by obtained ones by GC. Negative tests indicate safe drink in 100% of the samples that were confirmed in all cases by GC. This may be of great importance in prevention of toxic alcohol outbreaks worldwide.

## Data Availability

The datasets generated and/or analyzed during the current study are available from the corresponding author on request.

## Abbreviations

2,4-D: 2–4 dichlorophenoxyacetic acid
CA: Chromotropic acid
EMR: Eastern Mediterranean Region
EtOH: Ehanol
FID: Flame Ionization Detector
GC: Gas Chromatography
IQR: Inter Quartile Range
LMO: Legal Medicine Organization
Mn: Manganese
NPV: Negative Predictive Value
PPV: Positive Predictive Value
SD: Standard Deviation
SPSS: Statistical Package for the Social Sciences
